# NPC1 Deficiency Contributes to Autophagy-Dependent Ferritinophagy in HEI-OC1 Auditory Cells

**DOI:** 10.3389/fmolb.2022.952608

**Published:** 2022-07-22

**Authors:** Lihong Liang, Hongshun Wang, Jun Yao, Qinjun Wei, Yajie Lu, Tianming Wang, Xin Cao

**Affiliations:** ^1^ Department of Medical Genetics, School of Basic Medical Science, Nanjing Medical University, Nanjing, China; ^2^ Jiangsu Key Laboratory of Xenotransplantation, Nanjing Medical University, Nanjing, China; ^3^ Central Laboratory, Translational Medicine Research Center, The Affiliated Jiangning Hospital with Nanjing Medical University, Nanjing, China

**Keywords:** Niemann–Pick type C disease, NPC1 deficiency, hearing loss, NCOA4, ferroptosis

## Abstract

Niemann–Pick type C disease (NPCD) is a rare genetic syndrome characterized by cholesterol accumulation in multiple organelles. NPCD is mainly caused by gene deficiency of NPC intracellular cholesterol transporter 1 (NPC1). It has been reported that some of the NPCD patients exhibit clinical features of progressive hearing loss at high frequency and iron disorder, but the underlying relationship is unknown. A recent study has reported that ferroptosis contributes to the impairment of cochlear hair cells that are related to sensory hearing. In this study, we generated NPC1-deficient HEI-OC1 cells to show the effect of NPC1 deficiency on cochlear outer hair cells. We found that NPC1 deficiency enhances autophagy-dependent ferritinophagy to release Fe (II). Our work provides important insights into the effect of NPC1 deficiency in auditory cells, indicating that it induces ferroptosis and results in hearing loss.

## Introduction

Niemann–Pick type C disease (NPCD) is a genetic syndrome with a wide range of clinical features from rapidly fatal disorders in neonates to the onset of chronic neurodegenerative diseases in adults, affecting about 1/120,000 live births (Vanier, 2010). NPCD is mainly characterized by cholesterol accumulation, and it affects multiple organelles such as the central nervous system (CNS), visceral organelles, and auditory system (King, 2014a; Wheeler and Sillence, 2020). Current research show more interests on the former (Vanier, 2010; Santos-Lozano et al., 2015; Balboa et al., 2021; Van Hoecke et al., 2021), but fewer studies have focused on the effect of NPCD related to genetic hearing loss and the molecular mechanism has not been fully understood to date.

The gene deficiency of NPC intracellular cholesterol transporter 1 (NPC1) mainly contributes to NPCD, accounting for approximately 95% of the NPCD family (Vanier et al., 1996; Higgins et al., 1999). NPC1 is a multiple transmembrane protein, consisting of 3 large luminal domains (NTD, MLD, and CTD) and 13 transmembrane domains (TM), located on the late-endosomal/lysosomal (LE/L) membrane and functions as a cholesterol transporter from the lumen to the membrane of late endosomes/lysosomes (Gong et al., 2016; Qian et al., 2020). Besides the role in cholesterol regulation, NPC1 deficiency has also been reported to lead to the dyshomeostasis of multiple metals (Hung et al., 2014) and even cause a deficient immunoreactivity of ferritin (a protein for iron storage) in the liver and spleen (Christomanou et al., 1995; Christomanou and Harzer, 1996), suggesting an important role of NPC1 in iron regulation. Ferroptosis is a type of regulated cell death (RCD) driven by iron-dependent lipid peroxidation through inducing ROS generation by the Fenton reaction (Mou et al., 2019; Qu et al., 2022). Among the ferroptosis pathways, ferritinophagy is an autophagy-dependent process leading to ferritin-iron release (Gryzik et al., 2021). Recent studies have reported that ferroptosis contributes to the impairment of cochlear hair cells that relate to sensory hearing (Hu et al., 2020; Zheng et al., 2020). Moreover, some previous studies of NPCD have reported that NPC1 deficiency leads to metal dyshomeostasis (Hung et al., 2014) and lysosome dysfunction (Pluvinage et al., 2021; Roney et al., 2021) in multiple organelles. We thus hypothesize a possible mechanism of genetic hearing loss related to NPCD, that is, NPC1 deficiency causes outer hair cells (OHCs) loss or damage in the cochlea by inducing ferroptosis.

Here, we generated the NPC1^−/−^ HEI-OC1 cell line to simulate the auditory cells of NPCD patients. In this study, we found an altered autophagy flux in NPC1^−/−^ HEI-OC1 cells with increased autophagy synthesis and blocked autophagy degradation. We also found enhanced ferritin degradation and dysregulation of iron homeostasis in NPC1^−/−^ HEI-OC1 cells, which promote ferroptosis. Our study provides a novel insight into NPC1 function for further diagnosis and treatment of NPCD.

## Material and Method

### Cell Culture

HEI-OC1 and HeLa cells were preserved in our lab. HEI-OC1 cells were cultured in Dulbecco’s Modified Eagle’s Medium (DMEM, Gibco, United States) supplemented with 10% fetal bovine serum (#FSP500, ExCell Bio, China) in the cell incubator containing 10% CO_2_ at 33°C, and HeLa cells were cultured in DMEM supplemented with 10% fetal bovine serum, 100 U/mL penicillin, and 100 μg/ml streptomycin (#15140122, Gibco, United States) in the cell incubator containing 5% CO_2_ at 37°C.

### Chemical Compounds

Chloroquine (CQ, HY-17589A, 100 μM), wortmannin (#HY-10197, 1 μM), Mg132 (HY-13259, 10 μM), ferric ammonium citrate (FAC, #HY-B1645, 10 μg/ml), deferoxamine mesylate (DFOM, #HY-B0988, 100 μM), and Rapamycin (#HY-10219, 10 μM) were purchased from MCE (China).

### Generation of Stable NPC1-Deficient HEI-OC1 Cell Lines

The single-guide RNAs (sgRNAs) targeting the fifth exon of the mouse NPC1 gene (Supplementary Table S1) were designed on the CHOPCHOP website (http://chopchop.cbu.uib.no/) and ligated to digested PX330 plasmid containing the Cas9 backbone with T4 DNA ligase (Vazyme, China). The recombinant plasmids were transfected into HEI-OC1 cells by nucleofection (LONZA) with Amaxa™ Basic Nucleofector™ Kit, and the viable monoclonal cell colonies were obtained by G418 (#A1720, Sigma, Germany) screening and subjected to genotyping *via* direct PCR-based sequencing (Supplementary Table S2). The effectiveness of the NPC1-deficient cell lines was analyzed by the level of NPC1 protein and the expression of NPC1 mRNA.

### Total RNA Isolation and Quantitative Real-Time Polymerase Chain Reaction

Total RNA was extracted from the HEI-OC1 cells by the phenol chloroform extracting method, and cDNA was synthesized from 1 ug of the total RNA with the HiScript II One Step RT-PCR kit (Vazyme, China). Real-time PCR was performed using ChamQ SYBR qPCR Master Mix (Vazyme, China) on a Step One Plus Real-Time PCR System (Applied Biosystems, United States). The qRT-PCR primers are listed in Supplementary Table S3. Each sample was tested in triplicate, and the relative gene expression was obtained by the comparative CT method (2^−ΔΔCT^). The GAPDH served as the internal control.

### Western Blotting Analysis

The total protein obtained from the cells lysed with RIPA buffer (Beyotime, China) was separated by polyacrylamide gel electrophoresis (SDS-PAGE, Bio-Rad, United States) and then transferred onto polyvinylidene fluoride (PVDF) membranes (Merck, Germany). Blots were incubated in blocking buffer [5% skim milk powder (BD, United States) in PBS-T] for 2 h followed by incubation with the primary antibodies overnight at 4°C on a shaker. The membranes were probed with HRP-conjugated AffiniPure goat anti-mouse IgG (H+L) (1:5000, #SA00001-1, Proteintech, China) or HRP-conjugated AffiniPure goat anti-rabbit IgG (H+L) (1:5000, #SA00001-2, Proteintech, China) secondary antibodies for 2 h at room temperature, and the blot signals were visualized with a molecular imager ChemiDoc XRS+ imaging system (Bio-Rad) by supersensitive ECL chemiluminescent substrate (#BL520B-1, Biosharp, China).

### Filipin Staining

Cells were fixed with 4% paraformaldehyde for 15 min and then incubated with 1.5 mg/ml glycine for 10 min. After that, the cells were incubated in 50 μg/ml (PBS diluted) Filipin III ready-made solution (#SAE0087, Merck, Germany) for 2 h at room temperature. Images were acquired by laser confocal microscopy (LSM710, Zeiss) using 405 nm excitation wavelength.

### Free Cholesterol Content Assay

Free cholesterol content in HEI-OC1 cells was detected with the free cholesterol content assay kit (#BC 1890, Solarbio, China) according to the manufacturer’s instructions, as follows. The HEI-OC1 cells (5 × 10^6^) were harvested, and then 1 ml of ethanol was added. After ultrasonication and centrifugation, the supernatant was used as the solution to detect the free cholesterol content. The content of free cholesterol was calculated according to the formula given.

### Total Cholesterol Content Assay

The total cholesterol content in HEI-OC1 cells was detected with a total cholesterol content assay kit (#BC 1985, Solarbio, China) according to the manufacturer’s instructions, as follows. First, 1 ml of isopropanol was added into 5 × 10^6^ HEI-OC1 cells, and after ultrasonication and centrifugation, the supernatant was taken for detection. After incubation at 37°C for 15 min, the absorbance value at 500 nm was measured with a microplate reader. The absorbance value was then interpolated on a standard curve to obtain x value (standard curve: y = 0.5098x–0.0445). The total cholesterol content was calculated according to the formula given. TC (μmol/10000 cell) = 0.002x.

### Immunofluorescence Staining

Cells grown on the coverslips were fixed with 4% PFA for 15 min, then permeabilized with 0.1% Triton X-100 (Sigma-Aldrich, United States) for 10 min. After blocking with 10% goat serum for 60 min, the primary antibodies LC3A/B (1:200, #4108S, CST, United States), FTH1 (1:200, #4393S, CST, United States), FLAG (1:200, AE005, ABclonal, China), or HA (1:200, #5017S, CST, United States) was added to incubate with the cells overnight at 4°C, and the corresponding secondary antibodies—donkey anti-rabbit IgG Alexa Fluor 546 (1:1000, #A-21202, Thermo Fisher) and donkey anti-mouse IgG Alexa Fluor 488 (1:1000, #A10040, Thermo Fisher) for 2 h at room temperature. The nuclei were stained with DAPI (#F6057, Sigma). Images were acquired by laser confocal microscopy (LSM710, Zeiss).

### Electron Microscopy Analysis

Cells were collected and fixed with glutaraldehyde and osmic acid and dyed with 2% uranium acetate aqueous solution for 2 h. After gradient dehydration of alcohol, the cells were permeated with a mixture of acetone and embedding (1:1) for 2 h and then transferred to a pure embedding agent overnight. After embedding and polymerization, the cells were cut into sections at a thickness setting of 50 nm and stained with uranyl acetate and lead citrate, and photographed with an AMT CCD camera.

### Fe (II) Content Analysis

FerroOrange (#F374, Dojindo, Japan), a ferrous ion fluorescent probe, was used to detect intracellular ferrous ions. Cells were grown on the laser confocal dish or 24-well cell culture plates, were washed thrice with HBSS, and incubated with 1 μmol/l FerroOrange working solution for 30 min. Images were acquired by laser confocal microscopy or fluorescence microscope using 514 nm excitation wavelength.

### Intracellular Reactive Oxygen Species Level

The ROS detection kit (#KGT010-1, KeyGEN Biotech) was used for quantitative determination of intracellular reactive oxygen species levels based on the fluorescence intensity changes of DCFH-DA. The HEI-OC1 cells (5 × 10^5^) were seeded in a six-well plate and cultured overnight. DCFH-DA was diluted with DMEM at 1:1000 to reach a final concentration of 10 μM, then added into cells to incubate for 30 min in the dark. After washing thrice with DMEM, the cells were harvested and detected by flow cytometry using 488 nm wavelength. The ROSUP solution (50 μg/ml, 30 min) was used for the positive control.

### Construction of Overexpression Vector Plasmids

The cDNA extracted from the HEI-OC1 cells was used as a template for PCR amplification with KOD FX Neo (TOYOBO, Japan). The products were cloned into PXJ40-FLAG and PXJ40-HA, and renamed as FLAG-Fth1 and HA-NCOA4, respectively (Supplementary Table S4).

### Co-Immunoprecipitation

WT and NPC1^−/−^ HEI-OC1 cells were transfected with equal amounts of FLAG-Fth1 and HA-NCOA4 plasmids with lipofectamine 3000 reagent (#L3000150, Thermo Fisher) for 48 h. After the cells were lysed on ice with IP lysis buffer (Beyotime, China) and centrifuged, 20 μl of ANTI-FLAG® M2 Affinity Gel (#A2220, Sigma, United States) was added into the supernatant for immunoprecipitation at 4°C overnight on a shaker. After washing five times with IP lysis buffer, the immunoprecipitated materials were eluted with 20 μl of the protein loading buffer (6×), boiled for 10 min, and subjected to immunoblot analysis. The empty FLAG vector was used as the control.

### Cell Proliferation Assay

The CCK8 assay was used to determine the proliferation of HEI-OC1 cells. About 2000 cells were seeded in a 96-well plate and cultured overnight. Then 10 μl of the CCK8 reagent (#K1018, ApexBio) was added into the cells at the indicated time point, and the cells continued to be incubated for 2 h. The absorbance at 450 nm was then measured using a microplate reader. The OD values were used to plot the cell proliferation curve.

### Apoptosis Analysis

The HEI-OC1 cells were detected with the Annexin V-FITC/PI Apoptosis Detection Kit (#A211-01, Vazyme, China) to evaluate apoptosis levels. CCCP (#C2759, Sigma) was used to induce apoptosis. The detailed procedure is as follows: about 5 × 10^5^ cells were harvested by cell dissociation with EDTA-free trypsin, then the cells were washed twice with precooled PBS and followed by a resuspension with 100 μl of binding buffer (1×). After that, 5 μl Annexin V-FITC and 5 μl PI staining solution were added and incubation for 10 min in the dark. Next, 400 μl binding buffer (1×) was added in the end, and the samples were detected by flow cytometry within 1 h, with Ex: 488 nm.

### Intracellular Lipid Peroxides Detection

Liperfluo (#L248, Dojindo, Japan), a lipid fluorescent peroxide probe, was used to detect intracellular lipid peroxides. The cells in the six-well plate were washed with HBSS, and the final concentration of 5 μM Liperfluo working solution (DMEM diluted) was added into the cells. After incubation for 30 min, the cells were washed twice with HBSS and detected by flow cytometry, with Ex: 488 nm.

### Malondialdehyde Detection

Malondialdehyde, one of the lipid peroxide metabolites was detected by the malondialdehyde (MDA) colorimetric test kit (#E-BC-K028-M, Elabscience, China) according to instructions. The total protein concentration was determined using a BCA assay (#P0012S, Beyotime), and the MDA content was calculated by the given formula. MDA (nmol/mgprot) = ΔA1 / ΔA2 × 10 nmol/mL × f ÷ Cpr, where ΔA1 = OD value of sample - OD value of blank tube, ΔA2 = OD value of standard substance - OD value of blank tube, f is dilution ratio, and Cpr is protein concentration of sample (mgprot/mL).

### Statistical Analysis

All data are presented as mean ± SD. Student’s t-test was used for comparisons between two independent sample groups, one-way analysis of variance (ANOVA) was used for single-factor comparisons among multiple groups, and two-way ANOVA was used for two-factor comparisons among multiple groups; *p* < 0.05 was regarded as significant.

## Results

### Cholesterol Accumulated in NPC1-Deficient HEI-OC1 Auditory Cells

Besides CNS and visceral symptoms disorders, most NPCD patients exhibit a progressive high frequency hearing loss. The cochlea's OHCs are essential sensory receptor cells and are important for hearing in the organ of Corti, especially at high frequencies (Basch et al., 2016).

**FIGURE 1:**
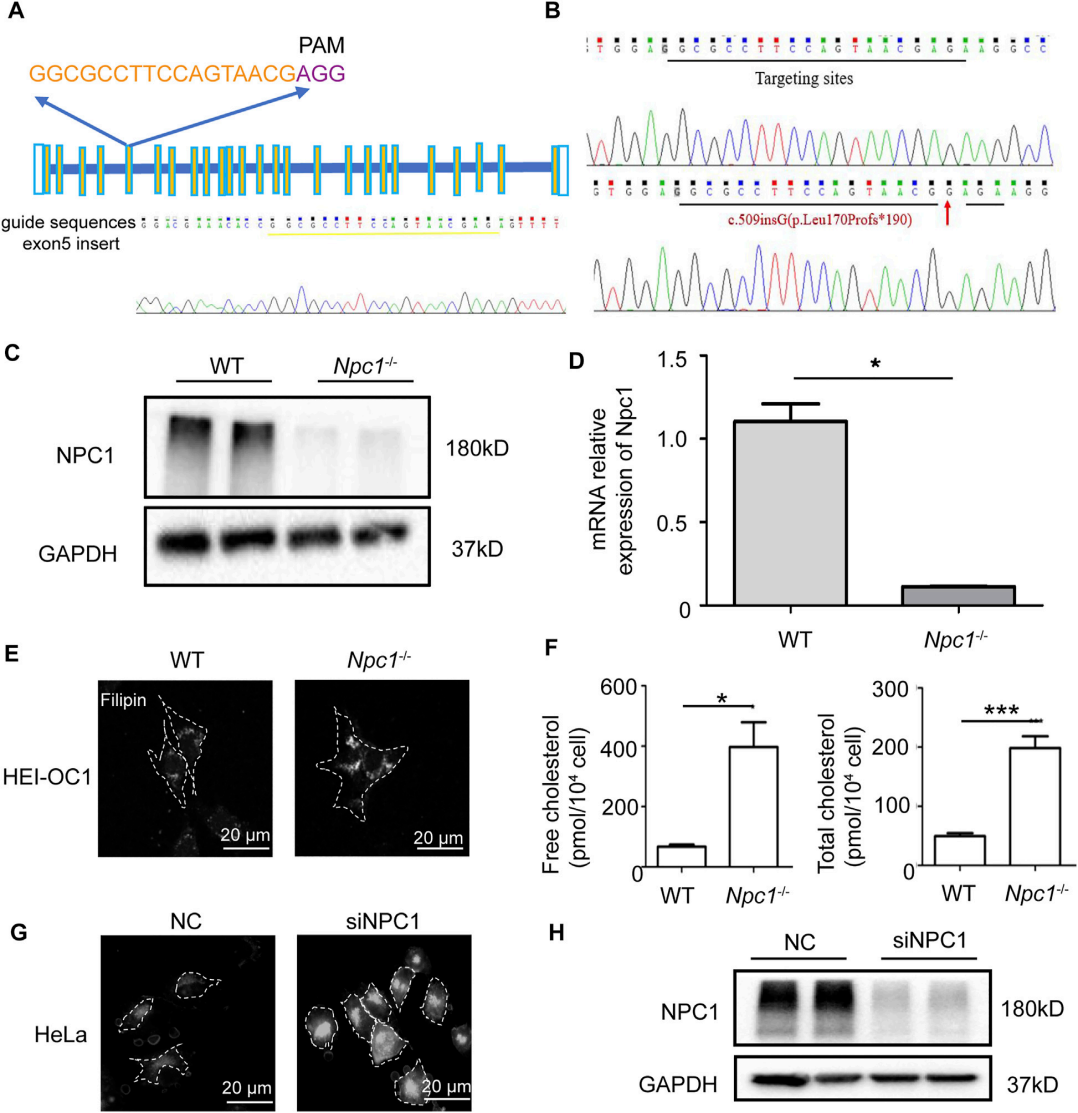
Establishment and validation of NPC1-deficient HEI-OC1 cell line. **(A)** The single-guide RNA (sgRNAs) targeting the fifth exon of the mouse NPC1 gene was designed on the CHOPCHOP website. The sgRNA is marked yellow and the PAM motif is marked purple. **(B)** The genotype of wild-type (WT) and NPC1 knockout (NPC1^−/−^) HEI-OC1 cells. Red arrow indicates mutant base location. **(C)** NPC1 protein detected by Western blot assay in the WT and NPC1^−/−^ HEI-OC1 cells. WT group are undisturbed HEI-OC1 cells. **(D)** NPC1 mRNA expression is determined by qRT-PCR in WT and NPC1^−/−^ HEI-OC1 cells. **(E)** Confocal images of filipin staining in the WT and NPC1^−/−^ HEI-OC1 cells. Scale bars, 20 μm. **(F)** Concentration of free cholesterol and total cholesterol in WT and NPC1^−/−^ HEI-OC1 cells. **(G)** Fluorescence images of filipin staining in control (NC) and NPC1 knockdown (siNPC1) Hela cells. Scale bars, 20 μm. NC group are HeLa cells treated with scrambled siRNA. **(H)** NPC1 protein detected by Western blot assay in control and NPC1 knockdown HeLa cells. GAPDH serves as the loading control. All data are from three independent experiments. Data are presented as mean ± SD values (*n* ≥ 3). *p < 0.05; ***p < 0.001.

To explore the effect of NPC1 deficiency in the auditory system, we thus generated NPC1-deficient HEI-OC1 auditory cells using the CRISPR-Cas9 gene-editing technique to truncate and alter amino acid sequences (Figures 1A–D). The single-guide RNA (sgRNA) targeting the fifth exon of the mouse NPC1 gene was designed and synthesized, then ligated to the PX330 vector plasmid with Cas9 skeleton protein (Figure 1A). The recombinant plasmid together with tdTomato was transfected into the HEI-OC1 cells by electroporation, and the viable monoclonal strains were screened by G418 prior to sequencing. The transfected cells underwent different degrees of gene editions, mainly manifested as changes in the number of bases which caused frameshift mutation and premature termination of protein translation (Figure 1B). The Western blot analysis results showed that the NPC1 band almost completely disappeared in the NPC1^−/−^ cells when compared with the wild-type (WT) cells (Figure 1C). The RT-PCR analysis results also showed that the NPC1 was significantly decreased in the NPC1^−/−^ cells when compared with the WT cells (Figure 1D).

Because cholesterol accumulation is the main feature of NPC1 deficiency previously reported in many other cell types, we first detected the cholesterol level in NPC1-deficient HEI-OC1 auditory cells. The cholesterol detection results showed that the cholesterol level in NPC1^−/−^ HEI-OC1 cells was much higher than in the WT cells, and the filipin staining images also showed cholesterol accumulation in NPC1^−/−^ HEI-OC1 cells (Figures 1E,F), which is similar to the results shown in the NPC1 knockdown HeLa cells (Figures 1G,H). We also found that transfecting NPC1 plasmid in NPC1^−/−^ HEI-OC1 cells could rescue the phenotype of cholesterol accumulation, while increasing NPC1 in the WT cells had no significant effect (Supplementary Figures S1A,B).

Our results show that we had generated NPC1^−/−^ HEI-OC1 cells successfully and also obtained a similar phenotype of cholesterol accumulation both in NPC1^−/−^ HEI-OC1 and NPC1 knockdown HeLa cells, indicating that the function of NPC1 had a good commonality between hearing cells and other cells in cholesterol regulation.

### NPC1 Deficiency Changed Autophagy Flux in HEI-OC1 Cells

Autophagy is a conserved catabolic process which plays an important role in cell fate determination and cell homeostasis under stress (Dikic and Elazar, 2018). It has been reported that high cholesterol stimulation induces autophagy (Li J, 2020), and autophagy also regulates cholesterol efflux (Ouimet et al., 2011).

**FIGURE 2:**
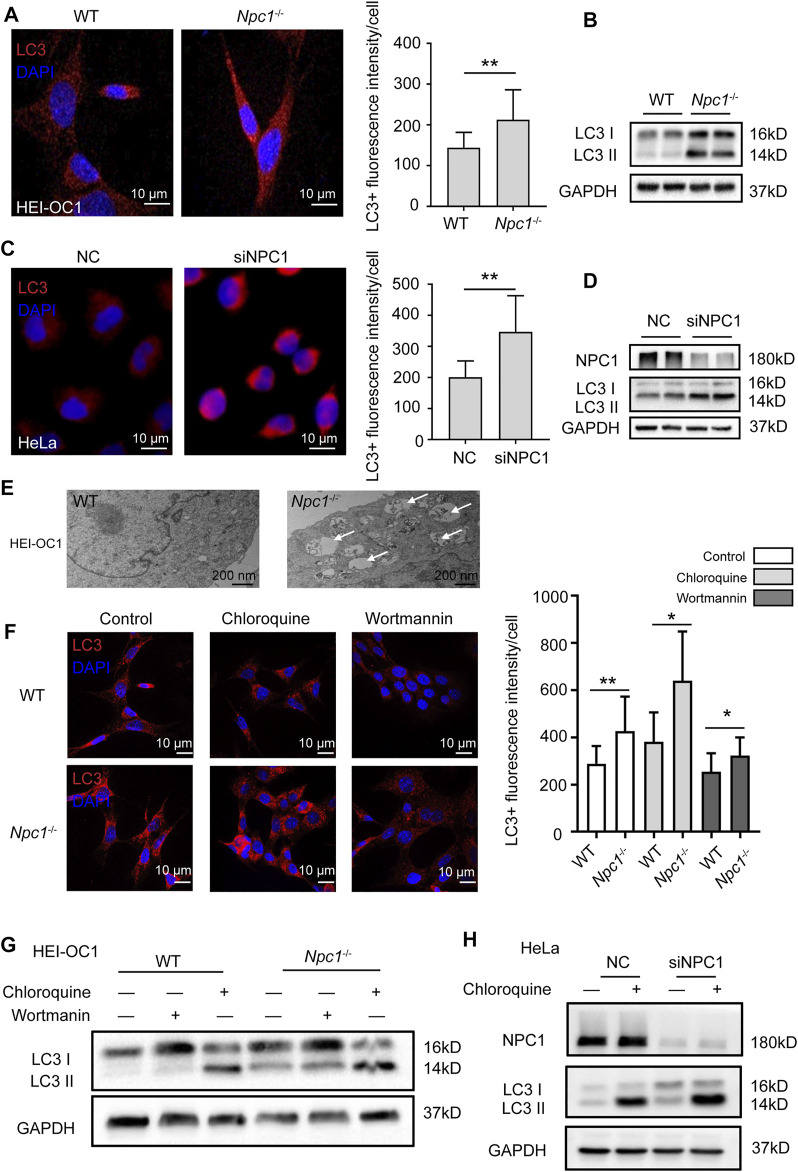
NPC1 deficiency enhanced autophagy flux. **(A)** Confocal images of immunofluorescence staining with anti-LC3 antibody in WT and NPC1^−/−^ HEI-OC1 cells. DAPI-labeled nucleus. Scale bars, 10 μm. **(B)** LC3 protein level detected by Western blot using the anti-LC3 antibody in WT and NPC1^−/−^ HEI-OC1 cells. GAPDH serves as the loading control. **(C)** Immunofluorescence images of control and NPC1 knockdown HeLa cells with anti-LC3 antibody. DAPI-labeled nucleus. Scale bars, 10 μm. **(D)** LC3 protein level detected in the control and NPC1 knockdown HeLa cells. GAPDH serves as the loading control. **(E)** TEM images of the WT and NPC1^−/−^ HEI-OC1 cells. Scale bars, 200 nm. White arrows indicate autophagosomes. **(F)** Immunofluorescence images of WT and NPC1^−/−^ HEI-OC1 cells with anti-LC3 antibody; chloroquine: 100 μM, 8 h; wortmannin: 1 μM, 8 h. Scale bars, 10 μm. **(G)** LC3 protein level detected by Western blot in WT and NPC1^−/−^ HEI-OC1 cells after the addition of chloroquine, 100 μM, 8 h or wortmannin, 1 μM, 8 h. GAPDH serves as the loading control. **(H)** LC3 protein level in control and NPC1 knockdown HeLa cells under the condition of chloroquine (10 μM). GAPDH serves as the loading control. Data are presented as mean ± SD values (*n* ≥ 3). *p < 0.05; **p < 0.01.

Due to the phenotype of cholesterol accumulation in NPC1-deficient HEI-OC1 cells shown earlier, we first detected the intracellular autophagy levels to confirm whether NPC1 deficiency induces autophagy in hearing cells. The immunofluorescent staining and Western blot results showed that the microtubule-associated protein 1 light-chain 3 (LC3II) expression level (an autophagy marker) was higher in NPC1-deficient HEI-OC1 cells than in WT cells, indicating an increase of autophagosomes in the NPC1-deficient HEI-OC1 cells (Figures 2A,B). We also observed increased LC3II levels in NPC1 knockdown HeLa cells (Figures 2C,D). By using the transmission electron microscope (TEM), we found more autophagosomes in NPC1^−/−^ HEI-OC1 cells (Figure 2E). Then, we transfected the NPC1 plasmid in the WT and NPC1^−/−^ HEI-OC1 cells to find out whether it could affect autophagy. We found that transfecting with NPC1 plasmid in NPC1^−/−^ HEI-OC1 cells could reduce LC3II, indicating an important role of NPC1 in autophagy (Supplementary Figure S1C).

We further evaluated the effect of NPC1 deficiency on autophagosome synthesis and degradation by treating the HEI-OC1 cells with autophagy inhibitors. To block the autophagosome degradation, we treated the HEI-OC1 cells with chloroquine (a late autophagy inhibitor) and found that the LC3II levels were increased and more pronounced in the NPC1^−/−^ HEI-OC1 cells, suggesting that NPC1 deficiency increased autophagy synthesis (Figures 2F–G). We next treated HEI-OC1 cells with wortmannin, another autophagy inhibitor, to determine the effect of NPC1 deficiency in autophagy degradation. As shown in Figures 2F,G, the wortmannin treatment significantly reduced the LC3II level in the NPC1^−/−^ HEI-OC1 cells, however, the LC3II levels in the NPC1^−/−^ HEI-OC1 cells were also higher than those found in the WT cells. A similar result was found in the NPC1 knockdown HeLa cells under the same treatment condition (Figure 2H).

Our data suggest that NPC1 deficiency changed autophagy flux by increasing autophagosome synthesis and blocking autophagosome degradation in HEI-OC1 cells.

### Abnormal Autophagy Causes Fe (II) Accumulation in NPC1-Deficient HEI-OC1 Cells

Iron is an important trace element that when present either in abnormal distribution or content can break redox homeostasis and affect normal physiological processes by generating toxic reactive oxygen species (ROS) through the Fenton reaction (Li K, 2020).

**FIGURE 3:**
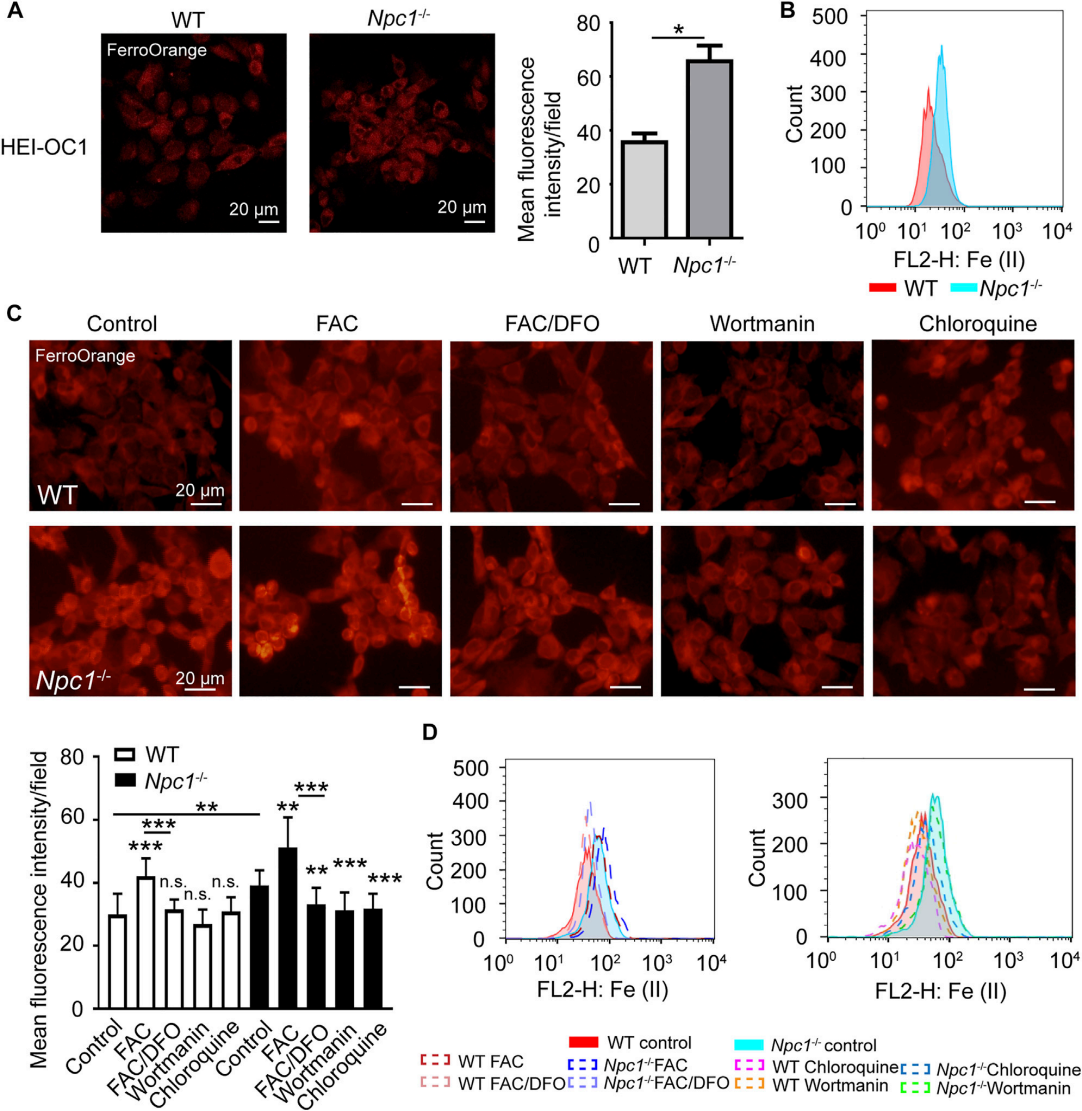
Fe (II) accumulation in NPC1-deficient HEI-OC1 cells. **(A)** Confocal images of Fe (II) stained with FerroOrange in WT and NPC1^−/−^ HEI-OC1 cells. Scale bars, 20 μm. *p < 0.05. **(B)** Flow cytometry detection images of Fe (II) in WT and NPC1^−/−^ HEI-OC1 cells. **(C)** Fluorescence images of intracellular Fe (II) stained with FerroOrange in WT and NPC1^−/−^ HEI-OC1 cells. FAC, 10 μg/ml, 24 h; DFO, 100 μM; wortmannin, 1 μM; chloroquine, 100 μM, 8 h. **(D)** Flow cytometry detection images of Fe (II) in WT and NPC1^−/−^ HEI-OC1 cells. FAC, 10 μg/ml, 24 h; DFO, 100 μM; wortmannin, 1 μM; chloroquine, 100 μM, 8 h. **p < 0.01; ***p < 0.001; ns: not significant.

A previous study had reported the failure of metal homeostasis in the liver and spleen from a mouse model of NPCD (Hung et al., 2014), and there have been many evidence about autophagy regulating iron content (Park and Chung, 2019), and we thus wondered whether NPC1 deficiency induced iron metabolic disorder in auditory cells through autophagy. We found out by fluorescence staining and flow cytometry detection that Fe (II) was increased in the NPC1^−/−^ HEI-OC1 cells (Figures 3A,B), indicating that NPC1 deficiency altered iron homeostasis in auditory cells. We also found that Fe (II) was not changed in the WT HEI-OC1 cells with NPC1 overexpression (Supplementary Figure S1D). Furthermore, we found that the Fe (II) fluorescence intensity level in the NPC1^−/−^ HEI-OC1 cells was higher than that in the WT cells with or without ferric ammonium citrate (FAC) treatment, while deferoxamine mesylate (DFO) could reduce the ferrous iron concentration in the HEI-OC1 cells (Figure 3C). In addition, we confirmed that Fe (II) was significantly reduced when the NPC1^−/−^ HEI-OC1 cells were treated with wortmannin or chloroquine (Figure 3C), suggesting that inhibiting autophagy by either repressing autophagosome synthesis or blocking autophagosome degradation could relieve the NPC1 deficiency–induced iron metabolic disorders.

Our results indicated that autophagy was involved in NPC1 deficiency–induced iron metabolic disorders.

### NPC1 Deficiency Promotes Ferritinophagy in HEI-OC1 Cells

The aforementioned data show that NPC1 deficiency increased Fe (II) in HEI-OC1 cells, and this alteration was regulated through an autophagy-dependent manner, and we thus focused on the underlying targets of autophagy in regulating Fe (II) content.

**FIGURE 4:**
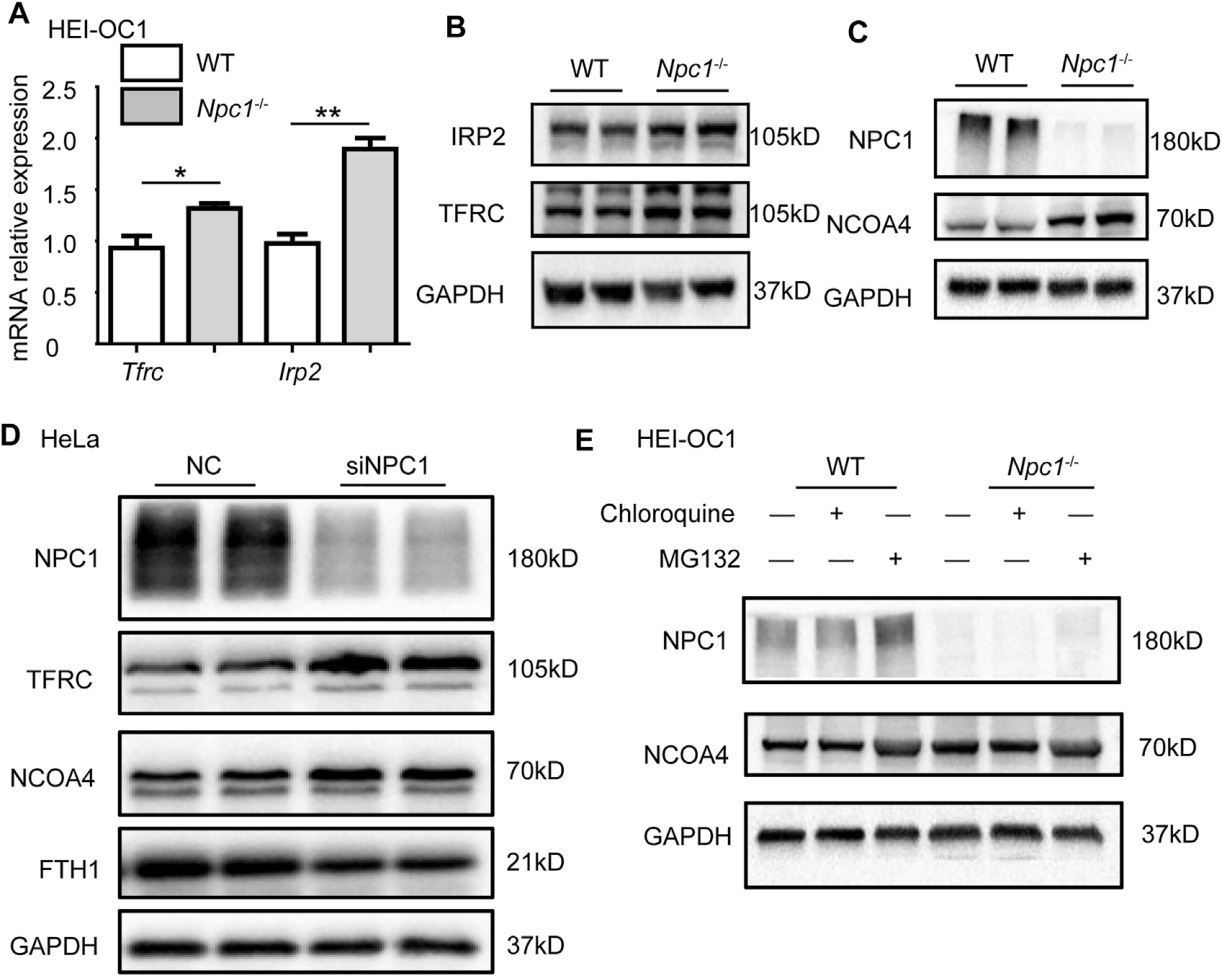
Abnormal iron flux in NPC1-deficient HEI-OC1 cells. **(A)** mRNA expression levels were determined by qRT-PCR in WT and NPC1^−/−^ HEI-OC1 cells. **(B,C)** The protein levels were detected by Western blot using antibodies to TFRC, IRP2, NPC1, NCOA4, and GAPDH in WT and NPC1^−/−^ HEI-OC1 cells. **(D)** HeLa cells transfected with control siRNA or siRNAs to NPC1 for 48 h, and the lysates subjected to immunoblotting using antibodies to NCOA4, TFRC, and FTH1. GAPDH serves as the loading control. **(E)** The NCOA4 protein level detected by Western blot under the condition of MG132 (10 μM, 8 h) or chloroquine (100 μM, 8 h) treatment in WT and NPC1^−/−^ HEI-OC1 cells using anti-NCOA4 antibody. Data are presented as mean ± SD values (*n* ≥ 3). *p < 0.05; **p < 0.01.

To confirm whether NPC1 deficiency affected iron intake, we first detected the expression levels of transferrin receptor protein 1 (TFRC) which plays an important role in iron intake and intracellular transport, and iron-responsive element-binding protein 2 (IRP2) which is an indicator of cellular iron bioavailability. We found that both TFRC and IRP2 were increased slightly in NPC1^−/−^ HEI-OC1 cells (Figures 4A,B), indicating that the iron intake and intracellular transport were slightly increased. To study the effect of NPC1 deficiency in iron release, we next detected the expression level of nuclear receptor coactivator 4 (NCOA4) which delivers ferritin heavy chain (FTH1) to the lysosome for iron release and is also considered to be a ferritinophagy marker. We found a significant increase in the NCOA4 expression level in NPC1^−/−^ HEI-OC1 cells (Figure 4C), indicating that NPC1 deficiency promotes iron release. A similar result was found in NPC1 knockdown HeLa cells, and we also found that FTH1, which is used for iron storage, was decreased (Figure 4D). The aforementioned results suggest that NPC1 deficiency promotes iron intake and iron release.

We next studied whether NPC1 deficiency contributed to autophagy-dependent ferritinophagy in the hearing sensory receptor cells. We found that repressing autophagy decreased the NCOA4 level, by treating the NPC1^−/−^ HEI-OC1 cells with chloroquine (Figure 4E). In addition, we found that MG132 (a reversible proteasome inhibitor) increased NCOA4 protein levels in both WT and NPC1^−/−^ HEI-OC1 cells, suggesting that the ubiquitin–proteasome degradation pathway was not influenced by NPC1 deficiency (Figure 4E). The aforementioned results suggest that the increased expression of NCOA4 induced by NPC1 deficiency was regulated through autophagy.

**FIGURE 5:**
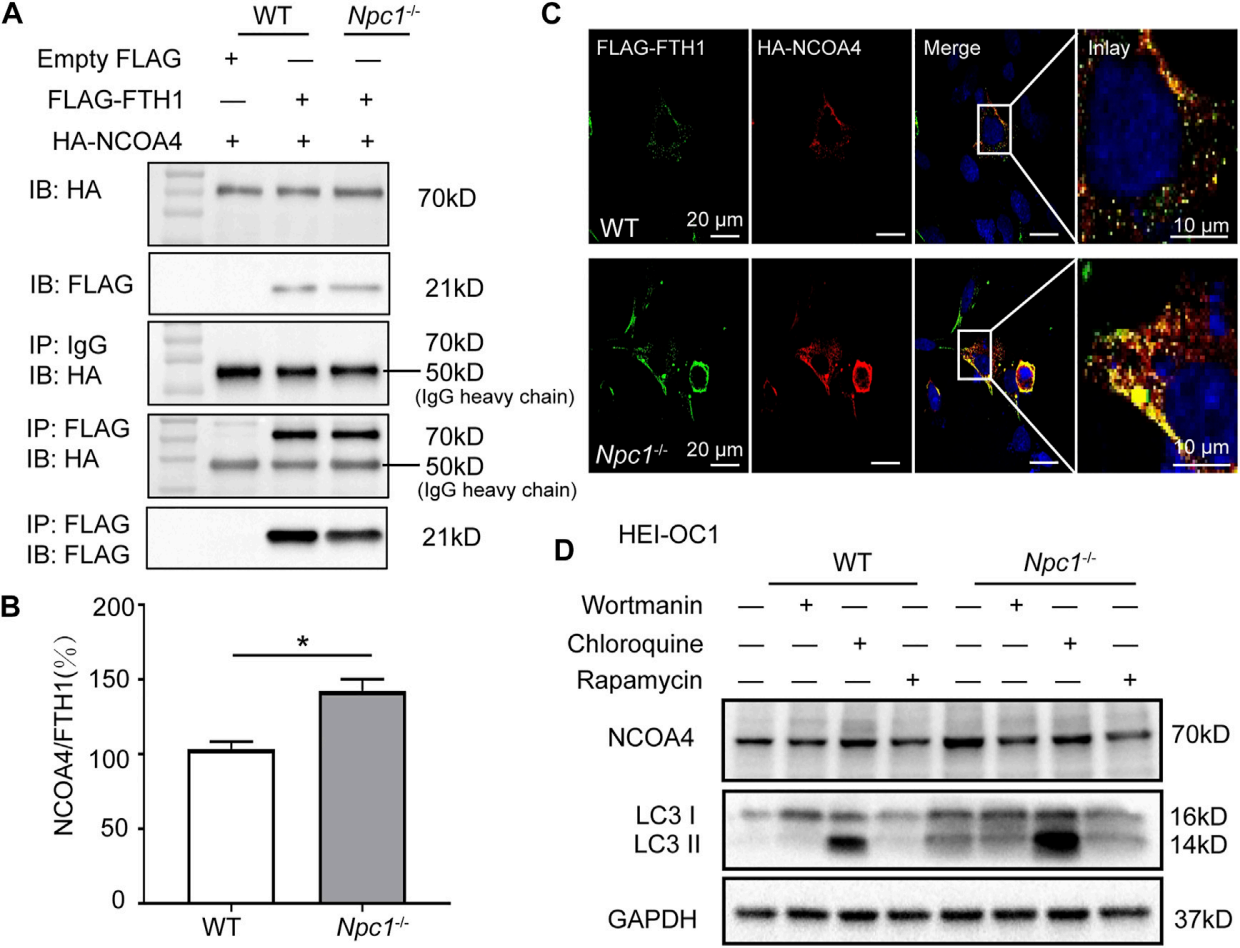
Enhanced ferritinophagy in NPC1-deficient HEI-OC1 cells. **(A)** Immunoblotting analysis performed to detect protein binding ability of FTH1 and NCOA4 in WT and NPC1^−/−^ HEI-OC1 cells. WT and NPC1^−/−^ HEI-OC1 cells transfected with equal amounts of FLAG-tagged Fth1 and HA-tagged NCOA4 plasmids for 48 h; samples then lysed and immunoprecipitated with anti-FLAG beads or anti-normal IgG. Inputs and immunoprecipitated fractions are detected by immunoblotting analysis with anti-FLAG and HA. The empty vector of the FLAG plasmid serves as the negative control. HA-NCOA4 serves as the loading control. **(B)** The protein binding ability normalized as the gray value ratio of HA to FLAG in **(A)**. **(C)** Confocal images of immunofluorescence staining with anti-FLAG and HA antibodies showing the colocalization of FTH1 and NCOA4 in WT and NPC1^−/−^ HEI-OC1 cells. Scale bar, 10 μm. **(D)** Immunoblotting analysis with anti-NCOA4 and anti-LC3 antibodies in WT and NPC1^−/−^ HEI-OC1 cells with or without treatment of wortmannin (1 μM, 8 h), chloroquine (100 μM, 8 h), or Rapamycin (10 μM, 8 h). GAPDH serves as the loading control. Data are presented as mean ± SD values (*n* ≥ 3). *p < 0.05.

We next transfected FLAG-tagged FTH1 plasmid and HA-tagged NCOA4 plasmid in HEI-OC1 cells to confirm whether NPC1 deficiency could promote NCOA4 to bind FTH1. The co-immunoprecipitation (co-IP) results showed that NCOA4 was increased in NPC1^−/−^ HEI-OC1 cells, suggesting that more NCOA4 bound to FTH1 in NPC1^−/−^ HEI-OC1 cells than they did in WT cells (Figures 5A,B). Furthermore, the immunofluorescent staining results showed an increase in colocalization between NCOA4 and FTH1 in NPC1^−/−^ HEI-OC1 cells (Figure 5C), supporting the aforementioned results, and we found that by treating the cells with either wortmannin or chloroquine to inhibit autophagy reduced NCOA4 expression levels (Figure 5D). However, inducing autophagy by treating the HEI-OC1 cells with Rapamycin, an autophagy inducer through inhibition of the mTOR pathway, did not reverse ferritinophagy in the NPC1^−/−^ HEI-OC1 cells, maybe due to the impaired function of the lysosome's outer membrane caused by NPC1 deficiency.

Taken together, our results show that NPC1 deficiency promoted autophagy-dependent ferritinophagy by enhancing the binding of NCOA4 to FTH1 and promoting it to release Fe (II).

### Enhanced Ferritinophagy Induces Ferroptosis in NPC1-Deficient HEI-OC1 Cells

Some previous studies have reported that inducing ferroptosis can inhibit HEI-OC1 cell proliferation, while treating the cells with a selective ferroptosis inhibitor liproxstatin-1 (Lip-1) could recover HEI-OC1 cell viability (Zheng et al., 2020).

**FIGURE 6:**
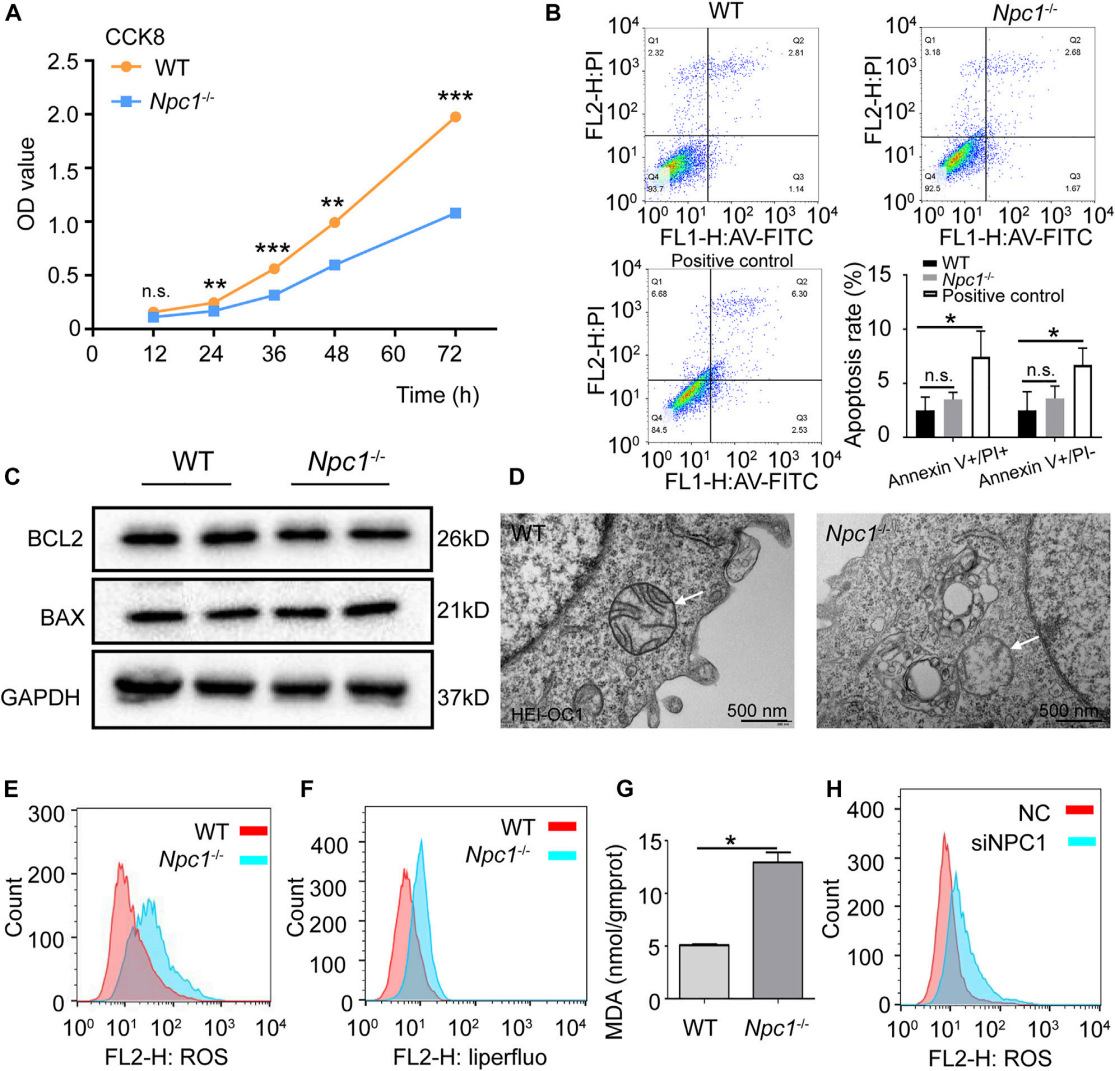
Ferroptosis induced by NPC1 deficiency in HEI-OC1 cells. **(A)** WT and NPC1^−/−^ HEI-OC1 cells detected with the CCK8 assay and the proliferation ability represented by OD values. **(B)** Annexin V-FITC/PI staining used to detect apoptosis of WT and NPC1^−/−^ HEI-OC1 cells by flow cytometry. WT cells treated with CCCP (10 μM) serves as the positive control. Ex: 488 nm. **(C)** Protein level of BCL2 and BAX detected by Western blot in WT and NPC1^−/−^ HEI-OC1 cells. GAPDH serves as the loading control. **(D)** TEM images of WT and NPC1^−/−^ HEI-OC1 cells; white arrow indicates mitochondrial outer membrane. **(E)** DCFH-DA fluorescent probe used to detect intracellular ROS in WT and NPC1^−/−^ HEI-OC1 cells by flow cytometry. **(F)** Liperfluo-fluorescent probe (5 μM) used to detect intracellular lipid peroxides in WT and NPC1^−/−^ HEI-OC1 cells by flow cytometry. **(G)** Content of MDA in WT and NPC1^−/−^ HEI-OC1 cells determined by the MDA detection kit. **(H)** HeLa cells transfected with control siRNA or siRNAs to NPC1 for 48 h and subjected to ROS detection with DCFH-DA fluorescent probe by flow cytometry. Data are presented as mean ± SD values (*n* ≥ 3). *p < 0.05; **p < 0.01; ***p < 0.001; ns: not significant.

We next confirmed whether ferritinophagy induced by NPC1 deficiency impacted the viability of auditory cells. The results of Figure 6A show that the proliferation of NPC1^−/−^ HEI-OC1 cells was inhibited and detected with the CCK8 assay. Inhibition of autophagy with chloroquine, but not wortmannin, could reverse the viability decrease seen in NPC1^−/−^ HEI-OC1 cells (Supplementary Figures S2A,B). To further confirm whether apoptosis contributed to cell damage, Annexin V/PI detection was performed with flow cytometry. We found that there was no significant difference between the WT and NPC1^−/−^ HEI-OC1 cells (Figure 6B). Moreover, the Western blot results showed that there was no significant difference between the WT and NPC1^−/−^ HEI-OC1 cells in BCL2 and BAX expressions (two apoptosis indicators) (Figure 6C), suggesting that the impaired proliferation of the NPC1^−/−^ HEI-OC1 cells was not caused through the apoptosis pathway.

To further confirm whether NPC1 deficiency induced ferroptosis in auditory cells, the TEM was used to observe the mitochondrial structure. We found reduced mitochondrial cristae and impaired outer mitochondrial membranes in the NPC1^−/−^ HEI-OC1 cells (Figure 6D), which are similar to the ferroptosis feature (Dixon et al., 2012). We also observed an accumulation of intracellular ROS in the NPC1^−/−^ HEI-OC1 cells (Figure 6E). In addition, lipid peroxides, an indicator of ferroptosis, were found increased in the NPC1^−/−^ HEI-OC1 cells, and MDA, which is one of the major metabolites of lipid peroxide, was increased in the NPC1^−/−^ HEI-OC1 cells (Figures 6F,G). We also found a similar trend of ROS in NPC1 knockdown HeLa cells (Figure 6H). All these results suggest that NPC1 deficiency induced ferroptosis in HEI-OC1 cells.

Taken together, our data show that 1) NPC1 deficiency changes autophagy flux by both increasing autophagosome synthesis and inhibiting autophagosome degradation; 2) abnormal autophagy induces ferritinophagy by promoting NCOA4 to deliver FTH1 to the lysosomes; 3) ferritinophagy promotes FTH1 to release Fe (II) and this is followed by an increase of lipid peroxide that induces ferroptosis in HEI-OC1 cells.

## Discussion

The mutation or absence of NPC1 is considered to be the main reason for NPCD occurrence, which is characterized as a lipid storage disorder with a high level of cholesterol that affects multiple organs and the CNS (Wheeler and Sillence, 2020). NPC1 deficiency is not only involved in CNS and visceral diseases but also in genetic hearing loss. The OHCs are important hearing sensory receptor cells in the auditory system and they convert the mechanical cilia swing caused by sound waves into electrical signals. Therefore, any loss or damage to the OHCs with their inability to regenerate thereby results in hearing loss (HL) (Kwan et al., 2009). In this study, we state a novel molecular mechanism of NPCD in causing genetic hearing loss, that is, NPC1 deficiency induces ferroptosis in auditory cells through an autophagy-dependent ferritinophagy manner.

The effect of NPC1 deficiency on autophagy flux provides a clue for follow-up research on ferritinophagy. Autophagy is associated with cholesterol regulation in many cell types. It is reported that high cholesterol induces autophagy in tendon-derived stem cells *via* the AKT/FOXO1 pathway (Li et al., 2020). In macrophage foam cells, autophagy mediates the generation of free cholesterol for ABCA1-dependent efflux (Ouimet et al., 2011). As an important cholesterol transporter, the studies of NPC1 related to autophagy have been previously reported in many cell types and mouse models. In primary human fibroblasts, NPC1 deficiency is reported to promote LC3II expression, which is to show an increase in active autophagy (Pacheco et al., 2007). In human dermal fibroblasts, the NPC1 deficiency impairs the clearance of autophagosomes induced by stored lipids (Elrick et al., 2012). In our data, we found that autophagosome synthesis was increased and degradation of autophagosomes was decreased in NPC1-deficient HEI-OC1 cells, which resulted in an altered autophagy flux in the auditory cells.

In this study, we further found that NPC1 deficiency increased Fe (II) content in auditory cells by enhancing the interaction between NCOA4 and FTH1, indicating the underlying molecular mechanism by which NPC1 regulates autophagy-dependent ferritinophagy. To date, there are only a few studies on NPC1 in the auditory system, and studies about the relationships in between NPC1, autophagy, and iron homeostasis are even fewer. Autophagy plays an important role in iron homeostasis, and it also induces ferritinophagy (Hou et al., 2016; Liu et al., 2020). It has been reported in lung fibroblastic cells that autophagy functions to promote the degradation of ferritin and TFRC expression to regulate iron content (Park and Chung, 2019). Nuclear receptor coactivator 4 (NCOA4) is crucial for ferritin-iron release by delivering ferritin for lysosomal degradation in ferritinophagy (Hou et al., 2016; Gryzik et al., 2021). A recent study has proved that disrupting the NCOA4-FTH1 protein–protein interaction by using compound 9a reduces intracellular Fe (II) and inhibits ferroptosis (Fang et al., 2021). In our results, we found that the expression levels of NCOA4 and TFRC were both increased, and NCOA4-FTH1 interaction was also enhanced in NPC1-deficient HEI-OC1 cells. Moreover, we found Fe (II) content and NCOA4 expression were regulated by autophagy, suggesting that NPC1 deficiency may cause autophagy-dependent ferritinophagy in auditory cells.

In addition, we found ferroptosis rather than apoptosis in NPC1-deficient HEI-OC1 cells. As we know, features of ferroptosis are different from those of apoptosis, with significant increase of Fe (II) content and exceeding lipid peroxide levels which are harmful to the mitochondria (Dixon et al., 2012; Wang et al., 2022). There have been some studies of NPCD on the effect of NPC1 deficiency and mental disorders. In a previous study of NPCD, it was reported that multiple metals such as Cu and Fe were found altered in the cerebellum and cerebrum of NPC1^−/−^ mouse (Hung et al., 2014). Recent research has also proven that iron content was increased in the brain of mice in an NPCD model (Hung et al., 2020). Besides the Fe (II) content, we also found that NPC1 deficiency changed cellular metabolic productions such as ROS, lipid peroxide, and MDA in HEI-OC1 cells. In addition, some researchers have shown progressive high frequency HL in NPC1 mutant mice (King, 2014b; Wheeler and Sillence, 2020), while others have also provided evidence of OHC loss in the basal region of the cochlea (Zhou et al., 2018). In our data, we have shown TEM images of the impaired mitochondrial membrane and provided evidence of the inhibited proliferation of NPC1^−/−^ HEI-OC1 cells, suggesting that NPC1 deficiency may impair auditory cells by ferroptosis.

Overall, this work reveals an underlying molecular mechanism by which NPC1 deficiency causes ferroptosis in auditory cells through the autophagy-dependent ferritinophagy pathway. This article presents a novel insight into the effect of NPC1 deficiency which may provide the potential target and therapeutic approach in the treatment of genetic hearing loss related to NPCD.

## Data Availability

The raw data supporting the conclusions of this article will be made available by the authors, without undue reservation.
