# Supplementary material for: NPC1 Deficiency Contributes to Autophagy-Dependent Ferritinophagy in HEI-OC1 Auditory Cells
Source: Front Mol Biosci. 2022 Jul 22;9:952608. doi: 10.3389/fmolb.2022.952608 (PMC9353266; doi:10.3389/fmolb.2022.952608)
Supplement: Supplementary file 1 [file Table1.DOCX]

**Supplementary Table 1 sgRNA oligonucleotide sequences targeting *Npc1***

| **Gene** | **Primer (**5’-3’**)** |
| --- | --- |
| Npc1- sgRNA -oligo1 | CACCGGCGCCTTCCAGTAACGAGA |
| Npc1- sgRNA -oligo2 | AAACTCTCGTTACTGGAAGGCGCC |

**Supplementary Table 2 Genotypes of *Npc1* knockout single cell clones**

| **Clone number** | **Genome sequence fragment** | **Gene type** |
| --- | --- | --- |
| **WT** | GTGATGTGGAGGCGCCTTCCAGTAACGAGAAGG | WT |
| **#08** | GTGATGTGGAGGCGCCTTCCAGTAACGGAGAAGG | +1bp |
| **#17** | GTGATGTGGAGGCGCCTTCCAGTAAC—AGAAGG | -1bp |
| **#60** | GTGATGTGGAGGCGCCTT—————CGAGAAGG | -7bp |

**Supplementary Table 3 Primer sequences for qRT-RCR**

| **Gene** | **Primer (**5’-3’**)** |
| --- | --- |
| M-q-*Gapdh*-F | TGGCCTTCCGTGTTCCTAC |
| M-q-*Gapdh*-R | GAGTTGCTGTTGAAGTCGCA |
| M-q-*Npc1*-F | ACCAAAACCCCTCCCAAAGG |
| M-q-*Npc1*-R | TGGCATTTCTCATGGGCTCC |
| M-q-*Tfrc*-F | ATGCCGACAATAACATGAAGGC |
| M-q-*Tfrc*-R | ACACGCTTACAATAGCCCAGG |
| M-q-*Ireb2*-F | TTCTGCCTTACTCAATACGGGT |
| M-q-*Ireb2*-R | AGGGCACTTCAACATTGCTCT |

**Supplementary Table 4 Primer sequences for overexpression vector**

| **Gene** | **Primer (**5’-3’**)** |
| --- | --- |
| FLAG-*Fth1*-F | CGGCCGCTCGAGTCTAGAACCACCGCGTCTCCC |
| FLAG-*Fth1*-R | GCGGGTTTAAACGGGCCCTTAGCTCTCATCACCGTGTC |
| HA-*Ncoa4*-F | TACGACGTGCCAGACTACGCAGGATCCAACACATCCCTGGAACAG |
| HA-*Ncoa4*-R | GTTCTGCTTTAATAAGATCTGGTACCTCACATCTGTAGAGGAGTTC |
