# Supplementary material for: NPC1 Deficiency Contributes to Autophagy-Dependent Ferritinophagy in HEI-OC1 Auditory Cells
Source: Front Mol Biosci. 2022 Jul 22;9:952608. doi: 10.3389/fmolb.2022.952608 (PMC9353266; doi:10.3389/fmolb.2022.952608)
Supplement: Supplementary file 2 [file DataSheet1.DOCX]

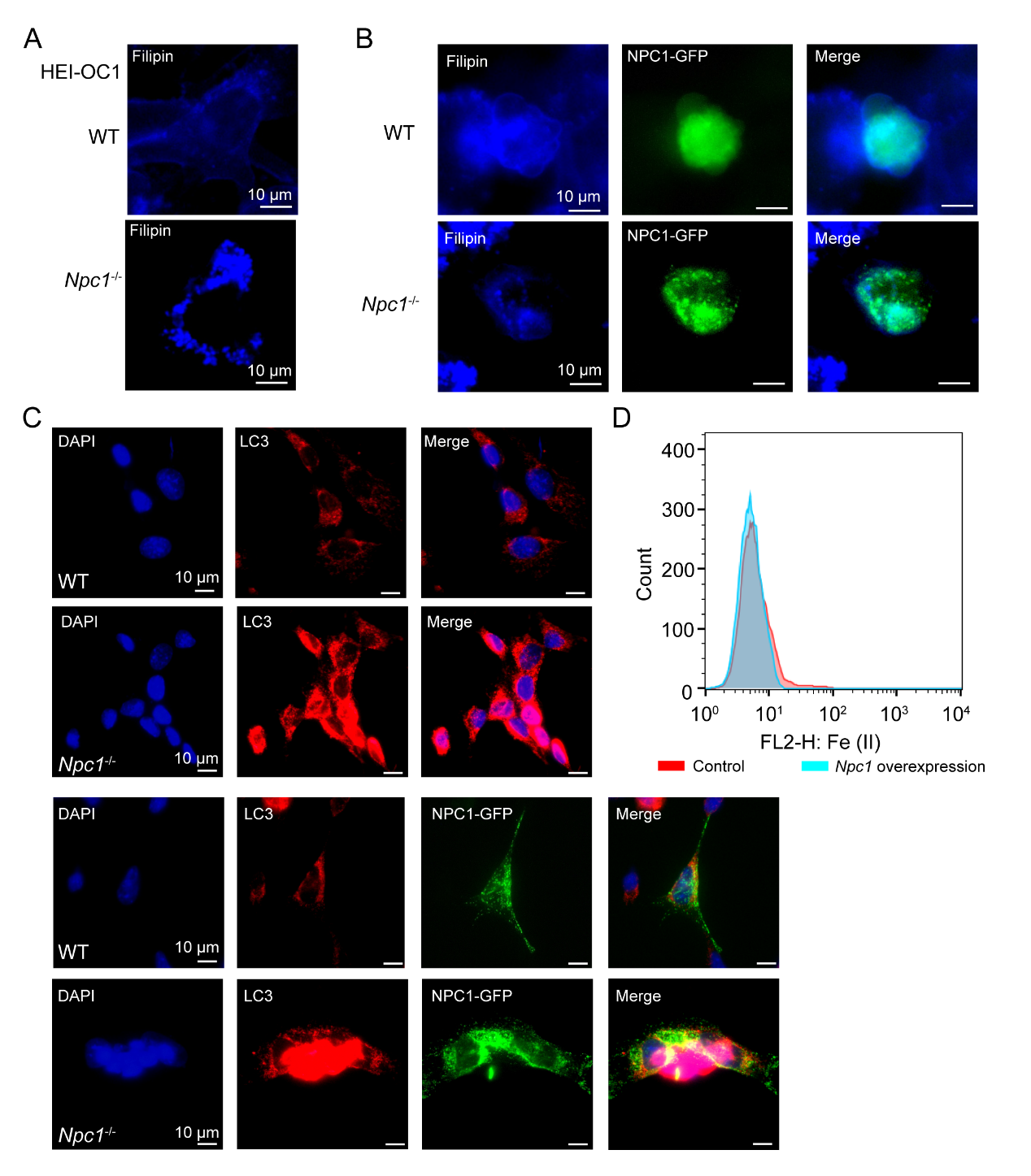


**Supplementary** **Figure 1 | Effect of NPC1 overexpression in HEI-OC1 cells.** Related to ***Figure 1-3****.* **(A)** Confocal images of filipin staining in the WT cells and *Npc1^-/-^* HEI-OC1 cells. Scale bars, 10 μm. **(B)** Confocal images of filipin staining in the WT cells and *Npc1^-/-^* HEI-OC1 cells transfected with *Npc1-GFP* plasmid. Scale bars, 10 μm. **(C)** Confocal images of immunofluorescence staining with anti-LC3 antibody in the WT cells and *Npc1^-/-^* HEI-OC1 cells transfected with or without *Npc1-GFP* plasmid. DAPI labeled the nucleus. Scale bars 10 μm. **(D)** Flow cytometry detection images of Fe (II) in the WT cells transfected with or without *Npc1-GFP* plasmid.


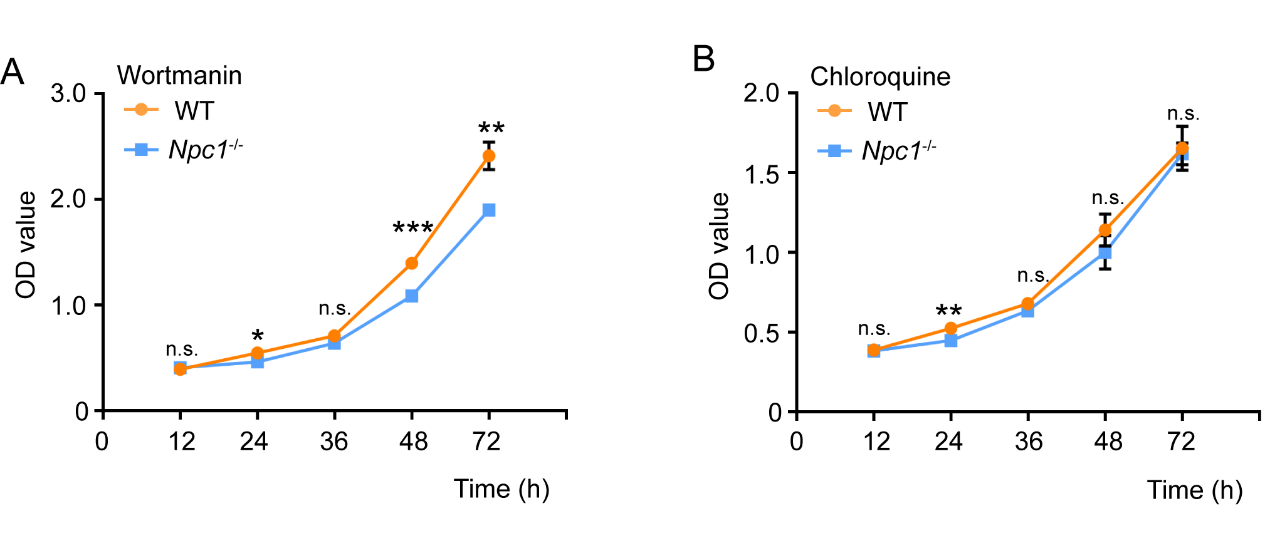


**Supplementary Figure 2 | Effect of autophagy inhibitors in HEI-OC1 cells.** Related to ***Figure 6****.* **(A)** The WT cells and *Npc1^-/-^* HEI-OC1 cells were treated with Wortmannin and then detected with CCK8 assay. The proliferation ability was represented by OD values. **(B)** The WT cells and *Npc1^-/-^* HEI-OC1 cells were treated with Chloroquine and then detected with CCK8 assay. The proliferation ability was represented by OD values. The data are presented as the mean ± SD values (n ≥ 3). **P* < 0.05; ***P* < 0.01; ****P* < 0.001; ns: not significant.
